# Variations in Composition, Antioxidant Profile, and Physical Traits of Goat Milk within the Semi-Intensive Production System in Mountainous Areas during the Post-Weaning to End-of-Lactation Period

**DOI:** 10.3390/ani13223505

**Published:** 2023-11-13

**Authors:** Eleni Kasapidou, Iraklis-Vasileios Iliadis, Paraskevi Mitlianga, Georgios Papatzimos, Maria-Anastasia Karatzia, Vasileios Papadopoulos, Michail Amanatidis, Vasiliki Tortoka, Ekaterini Tsiftsi, Antonia Aggou, Zoitsa Basdagianni

**Affiliations:** 1 Department of Agriculture, University of Western Macedonia, 53100 Florina, Greecevpapadopoulos@uowm.gr (V.P.);; 2 Department of Chemical Engineering, University of Western Macedonia, 50100 Kozani, Greecepmitliagka@uowm.gr (P.M.);; 3 Research Institute of Animal Science, HAO—Demeter, 58100 Giannitsa, Greece; karatzia@rias.gr; 4 School of Agriculture, Department of Animal Production, Aristotle University of Thessaloniki, 54124 Thessaloniki, Greecebasdagianni@agro.auth.gr (Z.B.)

**Keywords:** dairy goat, semi-intensive, lactation, chemical composition, fatty acid composition, antioxidant profile, physicochemical characteristics

## Abstract

**Simple Summary:**

Dairy products derived from mountain-origin milk are recognized for their superior composition, which can positively impact sensory and nutritional attributes. This study delves into the alterations in milk composition, nutritional quality, and antioxidant properties within the context of semi-intensive goat farming in mountainous regions, specifically during the post-weaning to end-of-lactation phase. The research was conducted in Greece, a region known for its prevalent mountain livestock farming systems. The study unearthed noteworthy fluctuations in milk yield and gross composition over the data collection period. The composition of milk fatty acids, particularly polyunsaturated fatty acids, exhibited a discernible correlation with forage intake, leading to enhanced nutritional lipid quality indices with prolonged grazing duration. Additionally, the study identified an upswing in total phenolic content towards the study’s conclusion, suggesting potential health benefits. Importantly, milk physicochemical properties remained relatively stable throughout the study, with no significant changes observed. In summary, this research offers valuable insights into the dynamics of milk composition and quality within semi-intensive goat farming systems situated in mountainous regions. These findings can prove invaluable to cheese manufacturers and farmers striving to elevate the quality of milk produced in mountainous regions under semi-intensive management systems.

**Abstract:**

Dairy products from mountain-origin milk are known for their superior composition and quality. This study aimed to examine changes in composition, nutritional quality, and antioxidant properties of milk from semi-intensively managed goats in mountainous regions during the post-weaning to end-of-lactation period. Bulk tank milk samples from 10 farms were collected bi-weekly in the period from March to September. The farms were situated in regions with an average altitude of 772.20 m above sea level. The results revealed significant variations in milk composition, with fluctuations in fat, protein, lactose, and total solids. Milk yield per doe showed seasonal differences, with the highest yield in April and the lowest in September. Fatty acid composition exhibited changes throughout the sampling period, with variations in polyunsaturated fatty acids. Nutritional indices, such as the atherogenicity index and thrombogenicity index, remained within the recommended values. Antioxidant properties, including total phenolic content, DPPH, FRAP, and ABTS, showed significant differences, with higher values toward the end of the study. Milk pH, electrical conductivity, brix value, and refractive index also exhibited variations, while density and freezing point remained relatively stable. The study provided valuable information that can be used to develop breeding and feeding plans to achieve uniform milk quality in mountainous regions.

## 1. Introduction

In Europe, the practice of mountain livestock farming systems, where animals are moved to highland pastures, is widespread. The transhumant pastoralism system prevails, involving the seasonal migration of ruminant herds from permanent valley farms to temporary farms at varying altitudes to exploit available grazing areas throughout the year [1,2,3]. This strategy is very important for the farmers because it supplements the annual forage budget while enabling access to public subsidies [4]. Moreover, it plays a crucial role in preserving traditional landscapes and biodiversity [5], while dairy products derived from mountain-origin milk exhibit better composition, affecting their sensory and nutritional qualities, compared to products from animals that are fed conserved forages or cereal-based concentrates [1,6]. These unique attributes substantiate the higher price associated with upland milk [7].

Although there is renewed interest in raising ruminants in upland areas due to the fact that exploitation with livestock does not compete with human food production [8], transhumance farming is declining [9] due to agricultural intensification [4]. As a result, many farmers are adopting the semi-intensive farming system, where animals are kept intensively during night and some part of the day and are moved to fenced or unfenced owned or rented pastures during some period of the day [10]. This system enables farmers to harness the benefits of grazing either uncultivated and/or semi-natural pastures, to enhance milk quality properties while also achieving higher milk yields, addressing a drawback of extensive production systems [5,11].

Small ruminant milk is a very important sector for European mountainous areas since approximately 30% of ewe and goat milk is produced in mountainous areas. Notably, in countries like Greece, nearly 39% of total ewe and goat milk originates from upland farms [12], predominantly destined for cheese production. The quality and composition of raw milk are among the major factors determining yield and quality of cheese while the chemical composition of goat milk changes significantly during lactation, resulting in variations in yield and sensory quality of the dairy products [13,14]. Globally, there is a rapid increase in dairy goat farming due to the rising demand for goat dairy products. In Greece, goat milk production has increased by almost 20% in the period 2016–2020 [15]. A limited number of studies have examined quality traits of goat milk produced in upland regions, primarily under the semi-extensive production system [16,17,18,19]. However, there has been a notable lack of comprehensive research on the milk composition resulting from semi-intensive management practices in farms situated in mountainous areas. This gap in knowledge is particularly significant, given the increasing trend among farmers to embrace the semi-intensive production system in recent times. The objective of the present study was to examine the changes in milk proximate composition, antioxidant profile, and physical characteristics of milk from commercial mountain semi-intensively managed goats throughout lactation and post weaning.

## 2. Materials and Methods

### 2.1. Sampling

**Figure 1:**
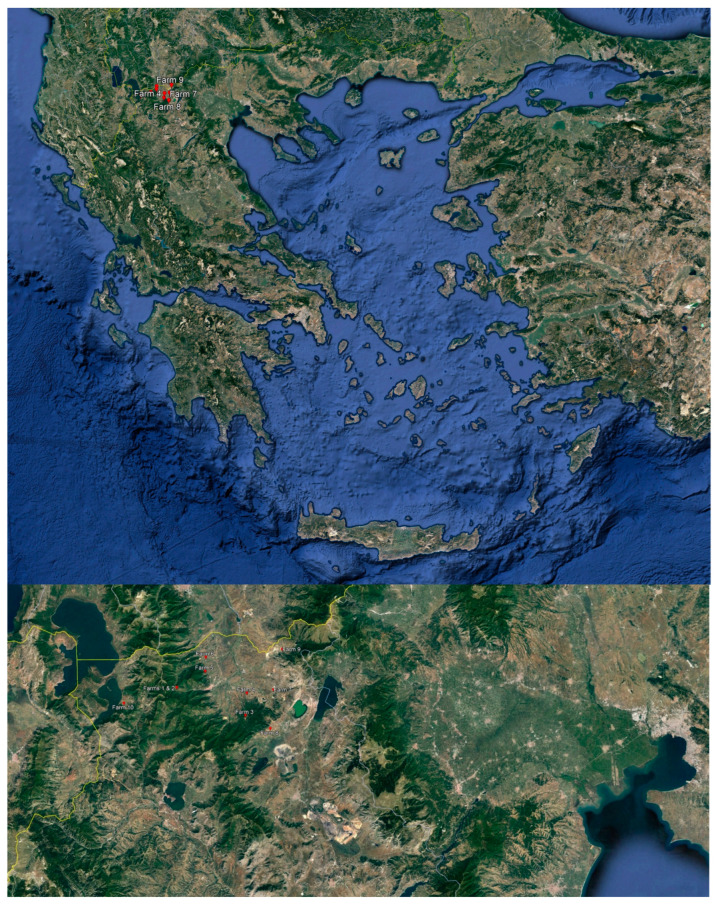
Locations of goat farms.

**Figure 2:**
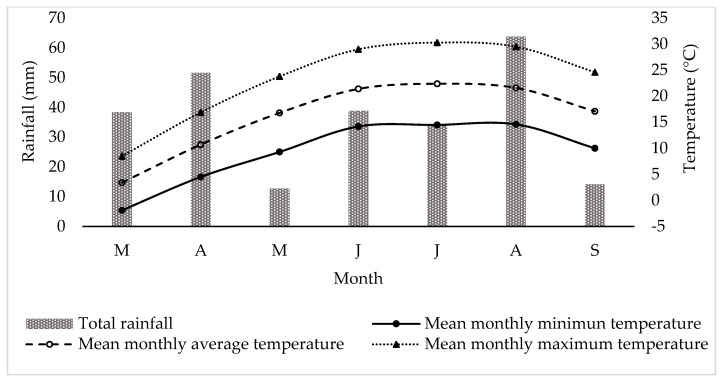
Rainfall and temperature conditions during the goat milk collection period (in order of appearance from left to right in Axis X: M, March; A, April; M, May; J, June; J, July; A, August; S, September).

The study was carried out in commercial goat farms located in the Regional Unit of Florina, Greece (40°46′58″ N 21°24′32″ E) in the period from March to September 2022 (Figure 1). The farms were located in areas designated as mountainous based on the topographic standards established by the Greek state. These criteria stipulate a minimum elevation of 800 m, which can be reduced to 600 m if the slope exceeds 16% [20]. The farms adhered to the semi-intensive management system, as described by the European Food Safety Authority [10]. Under this system, the animals were housed overnight and during milking, which occurred twice daily (early morning and late afternoon). The animals were moved to the pasture during daytime, in-between milking sessions. The goats were fed on a diet consisting of a combination of roughage, silage, concentrates, and grazing. The concentrates provided to the goats were sourced either commercially or through on-farm cultivation or a combination of both. Information about the farms and diets is presented in Table 1 and Table 2. Farms were selected on a convenience basis, taking into account the farmers’ willingness to participate in the study. Additionally, climatological data for the study period were obtained from the National Observatory of Athens for the weather station of Florina (https://penteli.meteo.gr/stations/florina, accessed on 28 October 2023) (Figure 2).

Bulk tank milk samples from 10 farms were collected bi-weekly after weaning when milk collection for the dairy processing plant began. The selected farms supplied their milk to a local dairy company producing cheese and yogurt. Samples were collected in 50 mL plastic screw-capped flasks, placed in isothermal portable containers with ice packs (≈4 °C) and transported to the laboratory. Upon arrival at the laboratory, the samples intended for analyzing total viable counts (TVCs), chemical composition, and determination of physicochemical characteristics were kept at 4 °C and analyzed within 24 h of collection. Samples aimed for the determination of fatty acid composition and antioxidant profile were kept at −20 °C until analyzed.

### 2.2. Milk Chemical Composition and Microbiological Evaluation

Milk composition (fat, protein, lactose, total solids, and solid non-fat content) was determined using a Milkoscan FT6000 Analyzer (Foss, Hillerød, Denmark). The microbiological assessment involved the determination of total viable counts (TVCs) using the Bactoscan FC (Foss Electric, Hillerød, Denmark) and the obtained data were log-transformed to normalize the distribution. 

### 2.3. Determination of Fatty Acid Composition

The milk samples were thawed overnight at 4 °C, and the next day, milk lipids were extracted using a chloroform/methanol solution (1:2 *v*/*v*) based on the method of Bligh & Dyer [21]. Solvents contained 0.01% (*wt*/*v*) of t-butyl-hydroxytoluene (BHT) to prevent oxidation of the unsaturated fatty acids during extraction. Fatty acid methyl esters were prepared from the extracted lipids by base-catalyzed methanolysis of the glycerides using methanolic KOH, according to the method ISO—IDF 15884 of the International Organization for Standardization [22]. The analysis of fatty acid methyl esters was carried out using an Agilent Technologies 6890N GC (Agilent Technologies, Inc., Santa Clara, CA, USA) equipped with a flame ionization detector (FID) and a 60 m × 0.25 mm i.d., 0.25 μm film thickness DB-23 (50% cyanopropyl, 50% dimethyl polysiloxane) capillary column (model number: Agilent 122-2362). The injector temperature was set at 250 °C. The oven temperature was programmed as follows: from 110 °C (held for 6 min) to 165 °C at 1 °C/min (held for 13 min), to 195 °C at 15 °C/min (held for 22 min), and finally to 230 °C at 7 °C/min (hold for 7 min). Helium was used as the carrier gas at a flow rate of 0.7 mL/min, and the injection volume was set at 3 μL with a split ratio of 1:50. The injection was performed using an Agilent 7683 Series auto-sampler. Fatty acids were identified using three commercial standard mixtures: (a) 37-component FAME mix (Supelco, 47885-U), (b) PUFA-2, animal source (Supelco, 47015-U), and (c) a mixture of cis- and trans-9,11- and -10,12-octadecadienoic acid methyl esters (Sigma, O5632-250MG) (Sigma-Aldrich, Taufkirchen, Germany) as reference standards. The fatty acid results are presented in percentages (%) of the total peak areas for all identified fatty acids. Fatty acids were categorized as saturated fatty acids (SFAs), monounsaturated fatty acids (MUFAs), and polyunsaturated fatty acids (PUFAs).

#### Nutritional Indices

The fatty acid profile was used to calculate nutritional indices related to healthy fat consumption. The applied indices are those reported in the recent review of Hanuš et al. [23] for the role of fatty acids in the nutritional value of milk. The polyunsaturated fatty acid/saturated fatty acid ratio was also calculated as it is the most frequently employed index for evaluating the nutritional quality of animal-derived foods [24].

Atherogenicity index:

$$\mathrm{AI}=\frac{C12:0+\left(4\times C14:0\right)+C16:0}{\Sigma MUFA+\Sigma n-6+\Sigma n-3}$$

Thrombogenicity index:

$$\mathrm{TI}=\frac{C14:0+C16:0+C18:0}{\left((0.5\times \Sigma MUFA)+(0.5\times \Sigma n-6PUFA)+(3\times \Sigma n-3PUFA)+(n-3/n-6)\right)}$$

Hypocholesterolaemic: hypercholesterolaemic fatty acid ratio (h/H):

$$h/H=\frac{C18:1n-9+C18:2n-6+C20:4n-6+C18:3n-3+C20:5n-3+C22:5n-3+C22:6n-3}{C14:0+C16:0}$$

Polyunsaturated fatty acid/ saturated fatty acid ratio:

$$\mathrm{PUFA}/\mathrm{SFA}=\frac{\Sigma \mathrm{PUFA}}{\Sigma \mathrm{SFA}}$$

Desaturation indices (Δ^9^ desaturase activity):

$${\mathrm{DI}}_{14}=\frac{C14:1\;cis9}{C14:0+C14:1\;cis9}\;{\mathrm{DI}}_{16}=\frac{C16:1\;cis9}{C16:0+C16:1\;cis9}\;{\mathrm{DI}}_{18}=\frac{C18:1\;cis9}{C18:0+C18:1\;cis9}$$

### 2.4. Total Phenolic Content and Antioxidant Profile

The total phenolic content (TPC) of the samples was determined using the Folin–Ciocalteau method [25]. The results are presented as milligrams of gallic acid equivalents (GAEs) per mL of milk. Free radical scavenging activity was assessed using the DPPH (2,2-diphenyl-1-picrylhydrazyl) method, following the protocol of Sanchez-Moreno et al. [26] with slight modifications. The results were expressed as μΜ of Trolox equivalents (TEs) per mL of milk, where Trolox is a water-soluble analogue of vitamin E: (6-hydroxy-2,5,7,8-tetramethylchroman-2-carboxylic acid). The reducing power activity of the samples was measured using the FRAP (ferric reducing antioxidant power) method, as reported by Pulido et al. [27] with minor changes. The results were expressed as μΜ of Trolox equivalents (TEs) per mL of milk. Finally, the total antioxidant capacity of the milk samples was determined using the ABTS [2,2′-azinobis-(3-ethylbenzothiazoline-6-sulfonic acid)] method, following the procedure outlined by Re et al. [28] with slight adjustments. The results were expressed as μΜ of Trolox equivalents (TEs) per mL of sample. Before measuring absorbance in all types of analyses, the samples underwent centrifugation to prevent any interference from other components. Calibration curves were prepared using gallic acid (GA) for TPC or Trolox for DPPH/FRAP/ABTS as standards, with concentrations varying from 0 to 1000 mg/mL and 0–1000 μM, respectively.

### 2.5. Milk Physicochemical Properties

Before analysis, samples were placed in a controlled temperature water bath to reach room temperature (20 °C) and following that they were thoroughly mixed by gentle inversion of the sample container multiple times without causing frothing. Milk pH was measured using a glass electrode with a built-in temperature sensor (5014T, Crison Instruments, Barcelona, Spain) in a pH-meter (GLP 21, Crison Instruments, Barcelona, Spain) which was calibrated with standard buffer solutions of pH 4.0 and 7.0 according to manufacturer’s instructions. The electrical conductivity of the samples was measured by a conductometer (GLP 31, Crison Instruments, Barcelona, Spain) using a Sodium Ion-Selective Electrode (50 70, Crison Instruments, Barcelona, Spain), calibrated with 147 μS/cm, 1413 μS/cm and 12.88 mS/cm buffer solutions. Refractive index and Brix value were determined using a digital refractometer equipped with a Peltier thermostat (DR6000-T, Krüss, Hamburg, Germany). Milk density was calculated from the Fleischman equation which takes into account the milk’s total solids and fat contents. Milk freezing point depression (FPD) was measured using MilkoScan FT 6000 equipment (Foss, Hillerød, Denmark).

### 2.6. Statistical Analysis

Data are arranged by calendar months and presented as monthly averages. The Levene test was employed to assess the homogeneity of variances. One-way analysis of variance was used, followed by Tukey’s post hoc test in cases of homogeneity of variances. In cases where variance homogeneity was not met, the Games–Howell test was applied to examine differences in milk contents throughout the study period. For all tests, statistical significance was considered when resultant *p*-values were <0.05. SPSS software (version 28.0, SPSS Inc., Chicago, IL, USA) was used for data analysis.

## 3. Results and Discussion

### 3.1. Farm and Flock Characteristics—Diets

The farms were situated in regions with an average altitude of 772.20 m above sea level, spanning from 618 to 989 m above sea level, meeting the criteria for being classified as mountainous areas [20]. The average count of lactating goats per farm was 151, with a range between 90 and 250 animals. According to Tsiouni et al. [29], the average goat farm size in Greece consists of 138 animals. Additionally, around 22% of the farms have more than 200 animals, as reported in another study [30]. The latter finding is in line with the results of the current study.

Throughout the collection period, the mean daily milk yield was 165.32 kg, varying from approximately 98 to 413 kg. Regarding the animal breeds in the flocks, five farms had animals from a single breed, either Damascus or Indigenous Greek (*Capra prisca*). Four farms had animals from different breeds, such as Damascus, Indigenous Greek, and Alpine, while one farm had crossbred animals from Damascus, Indigenous Greek, and Alpine breeds (Table 1). Pappa et al. [30] recently conducted a study profiling goat farms located in northwestern Greece. The study reported a similar trend, wherein approximately 55% of the examined farms kept single-breed animals, while the remaining 45% had mixed-breed animals. Gelasakis et al. [31] reported that the goats within Greek farms were either of native purebred breeds or resulted from crossbreeding with imported breeds, predominantly Damascus and Alpine.

**Figure 3:**
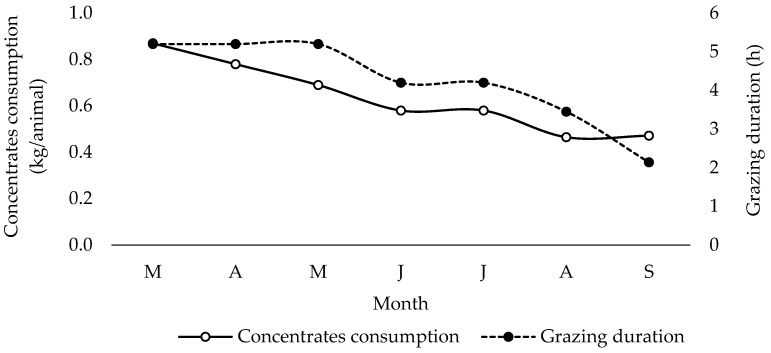
Changes in average daily intake of concentrates and grazing duration during the milk collection period (in order of appearance from left to right in Axis X: M, March; A, April; M, May; J, June; J, July; A, August; S, September).

The animals, on average, consumed 0.64 kg of concentrate feed per day (with a range of 0.55 to 0.89 kg), while the average grazing and browsing period lasted 4.28 h (ranging from 3.12 to 6.08 h) during the milk collection period (Table 2). The changes in the average daily intake of concentrates and grazing duration throughout the collection period are shown in Figure 3. As depicted in Figure 3, the decrease in supplementary feed consumption is attributed to grazing. Similarly, grazing duration is reduced during months like June, July, and August. Chebli et al. [32] conducted a study on grazing behavior and forage availability in mountainous areas. Their findings revealed that goats spent a greater amount of time grazing (eating) during the spring compared to summer and autumn. The researchers also noted that the biting rate (bites/min) was higher during the summer and autumn months, despite the lower forage availability and intake rate (gDM/ha) compared to spring.

Regarding supplementary feed, six farmers offered commercial feed, with two of them specifically providing feed designed for lactating goats. The remaining four farmers offered homemade blends of concentrated feed. The grazing material consisted mainly of shrubland while one farmer permitted his animals to graze on harvested corn and oat fields (Table 2).

### 3.2. Milk Yield and Composition—Milk microbiology

**Figure 4:**
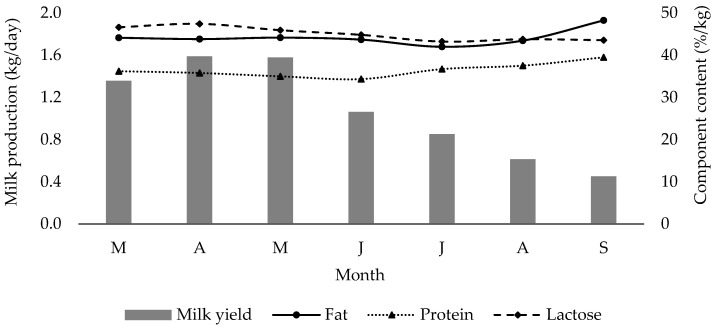
Changes in daily milk yield, protein, fat, and lactose contents during the collection period (in order of appearance from left to right in Axis X: M, March; A, April; M, May; J, June; J, July; A, August; S, September).

There were highly significant differences (*p* < 0.001) in milk yield at the farm and animal level and also in the gross composition of milk during the milk collection period (Table 3 and Figure 4). During the initial phase of milk collection (March, April, and May), higher levels of fat, protein, and solid non-fat were observed. However, these levels experienced a decline in June and July, followed by a subsequent increase towards the end of lactation. Lactose content exhibited high levels only at the onset of the post-weaning period, gradually decreasing until the end of the collection period. Soryal et al. [33] also reported similar fluctuations in the contents of fat, protein, lactose, and total solids in milk from Alpine goats receiving either a low or a high level of concentrates along with pasture grazing during lactation. Strzałkowska et al. [13] also noted variations in the chemical composition throughout the lactation period in goats that were fed on concentrates and hay in winter and on concentrates and pasture during the summer. Except for the fat content, which was lower, the levels of the other components fell within the reported range for goat milk, as shown in the recent study by Pappa et al. [30], which examined goat milk composition at the farm level. Data obtained from the Hellenic Agricultural Organisation [34] concerning the composition of goat milk produced in the Regional Unit of Florina demonstrated that the constituents of milk elements during the span of March to June 2022 were as follows: fat content was 4.58% in March and 4.86% in September, protein content was 3.63% in March and 3.79% in September, lactose content was 4.64% in March and 4.26% in September, while solid non-fat content was 9.23% in March and 9.28% in September.

Daily milk yield per doe was significantly affected (*p* < 0.001) during the sampling period with the highest yield observed in April and the lowest observed in September. A similar pattern was observed by Ataşoğlu et al. [35] in semi-intensively raised goats. According to Pulina et al. [36], the average milk yield per doe in Greece is estimated to be 250 L. Although, in the present study, the milk per doe was not recorded, an estimation based on farm yield and number of lactating animals leads to an approximate yield of 220 L during the milk collection period. An inverse relationship between the yield of protein and fat and milk yield was observed (Figure 3). The yield of fat and protein varied as a consequence of changes in milk yield and the content of both components. Ataşoğlu et al. [35] similarly observed changes in milk production, fat, and protein yield among semi-intensively raised goats over the course of lactation. These researchers adhered to Lock and Garnsworthy’s theory [37], which elucidated the decrease in fat and protein concentrations during the initial months of milk collection as a consequence of the dilution effect resulting from heightened milk output.

Highly significant differences (*p* < 0.001) were observed in the fat: protein ratio; nevertheless, the ratio remained consistently above one throughout the entire milk collection period (Table 3). According to Sandrucci et al. [38], when the milk fat content is lower than the protein content, it results to a reduction in cheese yield and a decline in quality attributes such as texture, smoothness, and fineness in goat cheeses [11]. These researchers also highlighted that the fat: protein ratio serves as a crucial indicator associated with the quality characteristics of milk intended for cheese production, which is also linked to management practices such as diet.

Lower levels (*p* < 0.05) of total viable counts (TVCs) were observed towards the end of lactation in relation to the post-weaning season. According to Lianou et al. [39], total viable count (TVC) can serve as an indicator of bacterial populations originating from multiple sources, including the udder skin as well as from the surfaces of milk-handling and storage equipment such as teat cups, milking parlor pipelines, and milk tanks. Additionally, it can also arise from the hands of milkers themselves, particularly in flocks or herds where hand-milking methods are employed. Total viable counts (TVCs) were lower than the values reported by Pappa et al. [30] (5.20–5.33 log cfu/mL) and Kondyli et al. [40] (6.05–6.14 log cfu/mL), suggesting that the hygiene conditions were notably better than those reported by the latter researchers.

### 3.3. Milk Fatty Acid Composition and Nutritional Value

Milk fatty acid composition either as individual fatty acids or as different lipid classes are presented in Table 4. Capric acid (C10:0), myristic acid (C14:0), palmitic acid (C16:0), and stearic acid (C18:0) were the predominant saturated fatty acids during the entire sampling period, whereas oleic acid (C18:1 *cis*-9) and linoleic acid (C18:2 n-6 *cis*) were the major monounsaturated and polyunsaturated fatty acid, respectively. Regarding saturated fatty acids, highly significant differences (*p* < 0.001) were found in the levels of caprylic acid (C8:0), capric acid (C10:0), lauric (C12:0), myristic acid (C14:0), and stearic acid (C18:0). However, this effect was not reflected in the levels of saturated fatty acids (SFAs) because there were no significant differences (*p* < 0.05) in the levels of palmitic acid (C16:0). The levels of saturated fatty acids such as caprylic acid (C8:0), capric acid (C10:0), lauric (C12:0), and myristic acid (C14:0) were gradually reduced throughout the sampling period, although there was an increase in the concentration of stearic acid (C18:0). The percentages of myristoleic (C14:1) and palmitoleic (C16:1) were significantly decreased (*p* < 0.01) during the examined period but no significant differences (*p* > 0.05) were found in the levels of total monounsaturated fatty acids (MUFAs). The contents of linoleic acid (C18:2 *n-6 cis*), and α-linolenic acid (C18:3 *n-3*) were also significantly affected (*p* < 0.01 and *p* < 0.001, respectively) throughout the entire study period. The levels of both fatty acids were gradually increased throughout the sampling period. Subsequently, the contents of polyunsaturated fatty acids (PUFAs) were also significantly increased (*p* < 0.001) during the sampling period. Ataşoğlu et al. [35], who had studied the changes in milk fatty acid composition during lactation in semi-intensively managed goats, reported a significant decrease in the levels of saturated fatty acids. On the other hand, Strzałkowska et al. [13] reported changes in the levels of different lipid classes during lactation and transition from winter diet (concentrates and hay) to summer diets (concentrates and fresh grass). In detail, the latter researchers reported a decrease in saturated fatty acids and an increase in monounsaturated and polyunsaturated fatty acids. Finally, Žan et al. [41] found a better fatty acid composition in milk from a mountain flock consisted of goats of Alpine breed grazing at an altitude of 1060–1075 m in comparison to milk from a highland flock consisted of goats of the Saanen breed grazing at an altitude of 615–630 m. In general, pasture feeding can affect milk fatty acid composition, but it is dependent on parameters such as the type of forage, variation in pasture availability, and stage of grass growth maturity [6,42]. The fatty acid composition during the entire study period is within the range reported by Kasapidou et al. [15] on retail goat milk on an annual basis. 

Regarding Δ^9^-desaturase activity (stearoyl-CoA desaturase), no differences (*p* > 0.05) were observed in C14:1/C14:0 and for C16:1/C16:0 while a highly significant difference (*p* < 0.001) was found in C18:1/C18:0 during the study period. C14:1/C14:0 is considered a reliable indicator to assess the effect of dietary changes on the Δ^9^-desaturase activity since myristic acid (C14:0) in the milk is produced by de novo synthesis in the mammary gland [37]. A higher Δ^9^-desaturase activity for C18:1/C18:0 was observed at the beginning of milk collection, and this is in agreement that there is an increase in the Δ^9^-desaturase activity in animals fed fresh grass i.e., spring and summer in cows [37]. Overall, there is limited understanding of the factors that influence Δ^9^-desaturase activity in goats. It is crucial to emphasize that the responses of milk fatty acid secretion and milk fat lipolysis to physiological and nutritional factors vary significantly between cows and goats [43].

The effect of forage plants and shrubs on goat milk fatty acid composition has been studied. For example, Alipanahi et al. [44] found that goats fed a diet containing oak acorn and extruded soybean seeds produced milk with a beneficial fatty acid composition for human health, whereas Ayeb et al. [45] reported that feeding oat hay can increase the content of unsaturated fatty acids in milk. However, in all of these studies, the effect of a single plant species was examined. In the present study, the animals were grazing on a variety of plant species along with supplementation with concentrates. Thus, comparing the results of the present study with other studies is inappropriate.

Changes in lipid quality nutritional indices during the period from March to September are presented in Table 5. Significant differences (*p* < 0.01) were found in the atherogenicity index whereas there were no changes in the thrombogenicity index (TI). The AI depicts the relationship between saturated fatty acids (SFAs), such as lauric (C12:0), myristic (C14:0), and palmitic acid (C16:0), that are considered pro-atherogenic and unsaturated fatty acids (UFAs) that are considered anti-atherogenic because they inhibit plaque formation and lower the levels of phospholipids, cholesterol, and esterified fatty acids. The TI refers to fatty acids’ thrombogenic potential, indicating their tendency to form clots in blood vessels [46]. In the present work, values of both indices did not exceed the recommended value (<3) [47] during the entire study period. The average AI and TI values are lower than those reported by Basdagianni et al. [48] for bulk tank milk samples collected during the entire lactation period (January to August) from commercial farms where the goats grazed in pastures located in semi-mountainous and mountainous regions in Greece. Barłowska et al. [49] also observed significantly lower AI and TI values in the milk of goats that grazed in mountainous regions during the day and received concentrate supplementation during milking. The values of both indices are lower than the ones reported by Kasapidou et al. [15] on retail goat milk during spring, summer, and autumn. 

Furthermore, sampling period did not affect (*p* > 0.05) the hypocholesterolaemic: hypercholesterolaemic (h/H) ratio, which is employed to represent the relationship between the hypocholesterolemic and the hypercholesterolemic fatty acids, and high values are desirable. It is worth noting that higher values of this ratio are considered desirable. Access to forage feeding did not affect the h/H ratio during the study period despite fluctuations in the average values. The h/H ratio values are higher than the ones reported in the literature [15,50].

Similarly, the polyunsaturated fatty acid/saturated fatty acid ratio (PUFA/SFA) was also not significantly altered throughout the milk collection period. The PUFA/SFA ratio is a commonly used indicator for evaluating the influence of diet on cardiovascular health. This index suggests that all polyunsaturated fatty acids (PUFAs) can reduce low-density lipoprotein cholesterol (LDL-C) and decrease overall serum cholesterol levels, while all saturated fatty acids (SFAs) tend to raise serum cholesterol levels. Consequently, a higher value of this ratio is associated with a more beneficial impact on cardiovascular health [24]. The PUFA/SFA ratio was far below the Department of Health’s recommendation of 0.45 [51] throughout the entire milk collection period. Generally, milk from ruminant animals tends to have a low ratio of polyunsaturated fatty acids (PUFAs) to saturated fatty acids (SFAs). According to the findings of Gibson et al. [52], who conducted a review of cohort studies, there is no consistent evidence suggesting that the consumption of dairy products is linked to an increased risk of cardiovascular disease. As grazing duration increased, higher PUFA/SFA ratio values were observed. The average PUFA/SFA value is comparable to that reported by Basdagianni et al. [48] for milk samples collected from commercial farms where goats grazed in semi-mountainous and mountainous regions.

Barłowska et al. [49] associated the increased levels of health-promoting fatty acids and the improved nutritional lipid quality parameters in milk obtained from goats grazing on natural mountain pastures with the richer floral diversity, specifically the greater variety of meadow plants and herbs present. 

### 3.4. Milk Total Phenolic Content and Antioxidant Profile

Table 6 displays changes in the milk total phenolic content and antioxidant profile during the sampling period. Highly significant (*p* < 0.001) differences were observed in the total phenolic content, which increased towards the end of the study, suggesting cumulative intake of phenolic compounds through grazing. The reported levels for total phenolic content are very versatile. Chávez-Servín et al. [53] reported 0.0879 mg GAE/mL in milk from animals fed on pasture, whereas Cabiddu et al. [54] reported total phenolic content ranging from 0.067 to 0.433 mg GAE/mL in animals raised on a combined stall fed and grazing system. The latter researchers found differences in the total phenolic content among April, May, and June and reported that the phenolic content of milk could be an interesting biomarker for milk produced under the extensive production system. Delgadillo-Puga et al. [55] found that grazing improved phenolic content in goat milk in comparison to milk from animals fed on concentrates. Amrit et al. [56] reported a seasonal variation in the phenol content in pasture grasses used for livestock production where higher levels were found in winter- and spring-grown pasture grasses. The higher level of total phenolic content observed in August and September are attributed to the greater period of intake via grazing that functions accumulatively. Differences in the total phenolic content between the present study and other studies are related to the different methodologies employed for the analysis. In the present study, milk samples were not subjected to protein precipitation as in the study of Vázquez et al. [57]. Polyphenols possess a significant binding affinity for proteins, potentially resulting in the creation of both soluble and insoluble complexes of protein–polyphenols [58].

With regard to the antioxidant activity, examined either as free radical scavenging activity (DPPH) or as ferric reducing antioxidant power (FRAP) or as total antioxidant capacity (ABTS), highly significant variations (*p* < 0.001–0.05) were observed throughout the milk collection period. Mal et al. [59] found that the stage of lactation did not affect the FRAP and ABTS values in milk from Gaddi goats, whereas significant differences were found in the DPPH radical scavenging activity (%). In detail, a higher activity was observed in the late lactation period in relation to the early and mid-lactation periods. However, the latter workers attributed this difference to the analytical methods employed. Chávez-Servín et al. [53] observed higher, but not significantly different, antioxidant activity, assessed as FRAP and DPPH, in milk from animals on a free ranging system in comparison to milk from animals kept indoors and fed on concentrates. Finally Delgado-Pertíñez et al. [18] in a similar study in mountain goats raised under the semi-extensive production system did not report any significant differences in ABTS values during lactation as examined in months from June to October. Di Trana et al. [60] reported that green forage-based diets improve the oxidative statuses of goats due to the elevated antioxidant contents of green grass such as α-tocopherol. Variations in antioxidant activity during the sampling period can be attributed to differences in the amounts of concentrates provided to the animals and their antioxidant content, which may be either endogenous and/or supplemented. Differences in the antioxidant activity can also be influenced by fluctuations in the levels of endogenous antioxidants, such as carotenoids, polyphenols, or α-tocopherol, within the grass species during the study period.

It is important to emphasize that the antioxidant activity was not uniformly expressed within the same sample because each assay operates through distinct mechanisms. To clarify, DPPH and ABTS rely on single electron transfer reactions, whereas FRAP operates on hydrogen atom transfer [61,62]. A direct comparison of the results of the current study with those from the literature is quite difficult because the results are expressed in different units (Trolox equivalent antioxidant capacity or percentage of radical scavenging). Additionally, different analytical procedures were employed.

### 3.5. Milk Physicochemical Characteristics

Changes in milk physicochemical characteristics during the collection period are presented in Table 7. Significant variations (*p* < 0.001) in milk pH were observed throughout the milk collection period. The milk pH gradually decreased and remained slightly higher than the value reported in the recent study by Pappa et al. [27] (6.66) throughout the entire sampling period. The milk pH was within the reported values (6.50–6.80) by Park et al. [63]. Foschino et al. [64] also documented a decline in pH during lactation in milking goats that were fed on grass, hay, and/or forage crops. They attributed this finding to the increase in protein concentration in milk towards the end of lactation, which was caused by the natural decline in milk yield. Milk pH affects rennet coagulation time since lower pH values are associated with shorter clotting time [65]. Regarding the correlation between milk pH and subclinical mastitis, Kandeel et al. [66] found that milk pH is not an effective screening method for mastitis in dairy cows, despite increases in milk pH due to the mixture of blood and extracellular fluid components with secreted milk.

Electrical conductivity was highly significantly (*p* < 0.001) affected during the examined period. Significantly higher values were observed June onwards in relation to electrical conductivity values recorded in the first three sampling months. Nevertheless, electrical conductivity values fall within the range reported in the review study by Park et al. [63]. According to Mabrouk and Petty [67], factors such as stage of lactation, season of the year, and feed can affect milk conductivity in cow milk. Milk’s conductive characteristics are associated with the presence of salts, primarily composed of chlorides, phosphates, citrates, carbonates, and bicarbonates of potassium, sodium, calcium, and magnesium. The relative concentrations of the various ions can vary as they are influenced by parameters such as animal breed, season of the year, feed, and stage of lactation. Fox et al. [68] reported that ions (particularly Na^+^, K^+^, and Cl^−^) are responsible for most of the electrical conductivity of milk, which is increased by the bacterial fermentation of lactose to lactic acid. In the present study, changes in the content of lactose as well as milk pH are reflected in the values of electrical conductivity. Voutsinas et al. [69] found significant changes in the concentrations of Na^+^, K^+^, and lactose in milk during lactation on Alpine goats that were kept indoors and fed on concentrates. Stergiadis et al. [70] also reported changes in the relative concentrations of milk minerals on an annual basis in retail goat milk samples. Milk electrical conductivity has been introduced as an indicator trait for mastitis detection in cows [71] because changes in lactose and mineral content during mastitic conditions influence electrical conductivity [72]. Nevertheless, Tangorra et al. [73] concluded that electrical conductivity is not a good mastitis indicator in dairy goats.

Both brix and refractive index values were affected (*p* < 0.05) throughout the examined period. Brix value and subsequently refractive index values can approximately provide information on the total solid content of milk [74]. The changes in both brix and refractive index values followed the same pattern of the changes in total solid content (Table 3). Application of portable refractometers is a rapid, nondestructive, precise, and cost-effective technique that enables farmers and small dairy producers to monitor the quality of milk, and in this respect, use of such equipment should be encouraged at the expense of an accurate estimation when necessary.

With regard to milk density, no significant differences (*p* > 0.05) were observed during the sampling period. Density was within the reported values given by Park et al. [63] (1.029–1.039 g/mL). A similar effect was reported by Kljajevic et al. [75], who had examined the effect of season in milk composition from Saanen goats. The density values of the milk exceeded 1.032 g/mL, which is the minimum value required by the Greek Food Legislation [76]. According to Peña-Avelino et al. [16], goat milk density is directly linked to goat milk components, mainly casein and fat, indicating that the content of these components did not vary significantly throughout the sampling period. The freezing point temperature was also not significantly affected (*p* > 0.05) during the sampling period. The freezing point temperature throughout the sampling period was within the range reported by Park et al. [63] (0.540–0.573 −°C) and Pappa et al. [30] (0.532–0.558 −°C). Higher freezing points were observed as the grazing duration increased. A similar trend was observed by Janštová et al. [77], who had related this effect with transition from winter to grazing. The latter authors also observed higher freezing point temperatures at the early months of lactation in relation to the end of lactation in White Shorthaired goats fed on summer diet (April–November) consisting of grazing, hay, and concentrates. Raynal-Ljutovac et al. [78] also reported that factors such as inadequate feeding, a significant proportion of seeds in the diets (as opposed to pasture or hay), diets lacking in roughage, an elevated protein-to-saturated fat ratio in stalling, reduced digestible sugars or energy, sodium chloride (NaCl) intake, water scarcity, and high temperatures can lead to alterations in the freezing point of cow milk. Elevated freezing point values are typically linked to the deliberate addition of water to increase the saleable milk quantities. However, since the monetary value of milk is based on fat and protein contents, this practice is infrequently utilized.

Unfortunately, there is a lack of research investigating the physicochemical attributes of milk from semi-intensively raised goats in mountainous regions. Such studies would have provided a basis for assessing the results of our current research.

## 4. Conclusions

This study provided valuable information on the changes in composition, nutritional quality, and antioxidant properties of milk from semi-intensively managed goats in mountainous regions during the post-weaning to end-of-lactation period. Significant differences in milk yield and gross milk composition were observed throughout the collection period. Forage intake affected milk fatty acid composition and particularly the content of polyunsaturated fatty acids. Regarding lipid quality, nutritional indices such as atherogonenicity index improved as grazing duration increased. Total phenolic content increased towards the end of the study period, whereas antioxidant activity, measured as DPPH, FRAP, and ABTS, showed significant variations throughout the collection period. Overall, milk physicochemical characteristics were not significantly affected during the study period.

Information regarding compositional changes in milk from semi-intensively raised goats in mountainous regions throughout the milk collection period can aid cheese manufacturers in crafting products with a desirable composition that aligns with the preferences of health-conscious consumers. Consequently, this can enhance product differentiation with respect to the milk production month.

Farmers can also use this knowledge to formulate initiatives aimed at minimizing fluctuations, such as developing breeding and feeding plans, as well as approaches for achieving uniform milk quality in such environments, thereby maximizing the positive impact associated with the semi-intensive production system on milk quality attributes.

## Figures and Tables

**Table 1:** Characteristics of each farm.

Farm No	Altitude (m)	Number of Goats in the Flock	Average Daily Milk Yield (kg)	Breed of Goats
1	989	120	110.00	Damascus
2	989	130	207.23	Damascus
3	777	200	149.58	Indigenous Greek (*Capra prisca*)
4	673	250	412.93	Indigenous Greek (*Capra prisca*), Alpine, Damascus
5	679	150	145.13	Cross breeds (Indigenous Greek (*Capra prisca*), Alpine, Damascus)
6	618	130	174.57	Indigenous Greek (*Capra prisca*), Damascus
7	732	150	122.23	Indigenous Greek (*Capra prisca*)
8	643	90	98.36	Indigenous Greek (*Capra prisca*), Alpine
9	753	150	108.80	Indigenous Greek (*Capra prisca*), Alpine, Damascus
10	869	140	126.38	Indigenous Greek (*Capra prisca*)

**Table 2:** Lactating goat diet information.

Farm No	Concentrates (Description)	Average Daily Intake (Concentrates) (kg)	Forage (Description)	Average Grazing Duration (h)
1	Commercial pellet for dairy goats	0.59	Oak, rowan, low bushes (kermes oak) wild vetch, tree spurge	4.23
2	Commercial pellet for dairy goats	0.65	Oak, rowan, low bushes (kermes oak) wild vetch, tree spurge	3.31
3	Corn, straw, grassland grasses	0.58	Oak, rowan, low bushes (kermes oak) wild vetch, tree spurge	3.42
4	Commercial concentrate	0.89	Corn stalks, oats	3.13
5	Commercial concentrate	0.57	Oak, rowan, low bushes (kermes oak) wild vetch, tree spurge	3.87
6	Commercial concentrate	0.64	Oak, rowan, low bushes (kermes oak) wild vetch, tree spurge	3.21
7	Corn, straw, grassland grasses	0.57	Oak, rowan, low bushes (kermes oak) wild vetch, tree spurge	5.08
8	Corn, straw, grassland grasses	0.66	Oak, rowan, low bushes (kermes oak) wild vetch, tree spurge	5.79
9	Commercial concentrate	0.55	Oak, rowan, low bushes (kermes oak) wild vetch, tree spurge	4.67
10	Corn, beans, straw, grassland grasses, alfalfa	0.67	Oak, rowan, low bushes (kermes oak) wild vetch, tree spurge	6.08

**Table 3:** Changes in milk production and composition during milk collection period.

Variable	Sampling Month							SEM	Significance
	March (n = 20)	April (n = 20)	May (n = 20)	June (n = 20)	July (n = 20)	August (n = 20)	September (n = 14)		
Farm milk yield (kg/day)	211.00 ^b^	246.10 ^b,c^	248.90 ^c^	163.50	130.50 ^a,b^	92.50 ^a^	68.57 ^a^	308.260	***
Fat (%)	4.28 ^a^	4.25 ^a^	4.28 ^a^	4.21 ^a^	4.05 ^a^	4.18 ^a^	4.64 ^b^	0.714	***
Protein (%)	3.51 ^a,b^	3.47 ^a,b^	3.39 ^a,b^	3.31 ^a^	3.54	3.61 ^b^	3.80 ^c^	4.602	***
Lactose (%)	4.52 ^c,d^	4.60 ^c,d^	4.45 ^c^	4.32 ^b,c^	4.17 ^a,b^	4.21 ^a,b^	4.19 ^a,b^	0.755	***
Total solids (%)	13.23 ^a^	13.15 ^a^	13.38 ^a,b^	14.70 ^c^	14.31 ^c^	15.18 ^c^	15.73 ^c^	4.305	***
Solid non-fat (%)	9.10 ^c^	8.82 ^b^	8.74 ^a,b^	8.52 ^a^	8.59 ^a,b^	8.69 ^a,b^	8.77 ^a,b^	0.846	***
Fat: protein ratio	1.22	1.23	1.26 ^c^	1.27 ^c^	1.16 ^a^	1.16 ^a,b^	1.23	0.206	***
TVC (log cfu/mL)	2.44 ^b^	2.41	2.37	2.32	2.34	2.26	2.13 ^a^	0.418	NS

SEM = standard error of the mean; NS = non-significant; *** = *p* < 0.001; superscripts ^a–d^ differ at *p* < 0.05.

**Table 4:** Milk fatty acid composition (% of total identified fatty acids) throughout the collection period.

Variable	Sampling Month							SEM	Significance
	March (n = 20)	April (n = 20)	May (n = 20)	June (n = 20)	July (n = 20)	August (n = 20)	September (n = 14)		
Fatty acid									
C4:0	1.16	1.23	1.25	1.25	1.33	1.29	1.17	0.272	NS
C6:0	1.57	1.68	1.63	1.58	1.61	1.62	1.52	0.209	NS
C8:0	2.29 ^a,b^	2.42 ^b^	2.32 ^a,b^	2.17 ^a^	2.14 ^a^	2.18 ^a^	2.15 ^a^	0.458	***
C10:0	9.32 ^b^	9.12 ^b^	8.63	8.07 ^a^	8.03 ^a^	8.19 ^a^	8.52	2.264	***
C11:0	0.17	0.19	0.19	0.33	0.17	0.29	0.12	0.310	NS
C12:0	4.84 ^c^	4.03 ^b^	3.68 ^a,b^	3.20 ^a^	3.41 ^a,b^	3.51 ^a,b^	4.12 ^b^	2.438	***
C13:0	1.22	1.24	1.16	1.57	1.06	1.39	0.84	0.958	NS
C14:0	10.66 ^b^	9.09 ^a^	8.95 ^a^	8.37 ^a^	9.23 ^a^	9.06 ^a^	9.91 ^a,b^	3.257	***
C14:1	0.40 ^b^	0.30 ^a^	0.27 ^a^	0.31 ^a^	0.29 ^a^	0.33 ^a,b^	0.33 ^a,b^	0.190	***
C15:0	0.93	0.84	0.88	0.90	0.93	0.88	0.87	0.143	NS
C15:1	0.27	0.25	0.24	1.60	0.23	1.44	0.19	2.743	NS
C16:0	28.13	25.62	25.33	25.10	26.92	25.59	27.10	4.980	NS
C16:1	0.41 ^c^	0.40 ^b,c^	0.38	0.38	0.34	0.34 ^a,b^	0.31 ^a^	0.154	**
C17:0	0.68 ^a^	0.70 ^a^	0.78	0.84 ^b^	0.86 ^b^	0.77	0.75	0.295	***
C17:1	0.30	0.25	0.24	1.00	0.27	0.92	0.25	1.509	NS
C18:0	9.02 ^a^	11.95 ^b,c^	13.90 ^b,c^	13.57 ^b,c^	13.58 ^b,c^	12.44 ^c^	12.12 ^c^	7.439	***
C18:1 *trans*	0.89	1.09	0.94	0.96	1.04	0.89	0.90	0.340	NS
C18:1 *trans-11* (VA)	0.71	1.15	1.12	2.21	1.08	2.17	0.95	2.623	NS
C18:1 *cis*-9	23.51	23.99	22.86	21.42	22.94	22.23	23.41	3.785	NS
C18:2 *n-6 trans*	0.55	0.90	0.92	0.96	0.67	0.77	0.64	0.694	NS
C18:2 *n-6 cis*	1.92	1.99	2.31 ^b^	2.20	2.09	1.94	1.80 ^a^	0.738	**
C18:3 *n-3*	0.64	0.84	1.23	1.28	1.12	0.94	1.09	0.991	***
C18:2 *cis-9 trans-11* (CLA)	0.79	0.94	0.99	0.97	0.84	1.00	0.99	0.311	NS
Lipid class									
SFA ^1^	69.99	68.09	68.70	66.94	69.29	67.21	69.19	4.945	NS
MUFA ^2^	26.49	27.42	26.04	27.87	26.19	28.32	26.34	3.989	NS
PUFA ^3^	3.52 ^a^	4.49 ^b^	5.26 ^d^	5.19 ^c,d^	4.52 ^b,c^	4.47 ^b^	4.47 ^b,c^	2.559	***
UFA ^4^	30.01	31.91	31.30	33.06	30.71	32.79	30.81	4.945	NS
OCFA ^5^	3.40	3.28	3.30	5.91	3.35	5.41	2.90	5.193	NS
n-3	0.57 ^a^	0.84 ^b^	1.23 ^c^	1.28 ^c^	1.12 ^c^	0.94 ^b^	1.09 ^b,c^	1.097	***
n-6	2.47 ^a^	2.89	3.23 ^c^	3.04 ^b,c^	2.77	2.62 ^a,b^	2.46 ^a^	1.251	***
Δ^9^-desaturase activity									
DI_14_	0.04	0.03	0.03	0.07	0.03	0.07	0.03	0.078	NS
DI_16_	0.01	0.02	0.01	0.05	0.01	0.04	0.01	0.066	NS
DI_18_	0.72 ^c^	0.67 ^b,c^	0.62 ^a,b^	0.60 ^a^	0.63 ^a,b^	0.63 ^a,b^	0.66 ^b^	0.180	***

^1^ = saturated fatty acids; ^2^ = monounsaturated fatty acids; ^3^ = polyunsaturated fatty acids; ^4^ = unsaturated fatty acids; ^5^ = odd chained fatty acids; SEM = standard error of the mean; NS = non-significant; ** = *p* < 0.01; *** = *p* < 0.001; superscripts ^a–d^ differ at *p* < 0.05.

**Table 5:** Milk fat nutritional indices throughout the collection period.

Index	Sampling Month							SEM	Significance
	March (n = 20)	April (n = 20)	May (n = 20)	June (n = 20)	July (n = 20)	August (n = 20)	September (n = 14)		
AI ^1^	2.60 ^b^	2.13 ^a^	2.14 ^a^	2.06	2.25	2.22	2.41	0.815	**
TI ^2^	2.95	2.61	2.59	2.52	2.74	2.70	2.74	0.621	NS
h/H ^3^	0.69	0.80	0.80	1.19	0.75	0.96	0.73	0.751	NS
PUFA/SFA ^4^	0.05 ^a^	0.07 ^b^	0.08 ^b^	0.08 ^c^	0.07 ^b^	0.07 ^b^	0.06 ^b^	0.041	***

^1^ = atherogenicity index; ^2^ = thrombogenicity index; ^3^ = hypocholesterolaemic: hypercholesterolaemic ratio; ^4^ = polyunsaturated fatty acid/saturated fatty acid ratio; SEM = standard error of the mean; NS = non-significant; ** = *p* < 0.01; *** = *p* < 0.001; superscripts ^a–c^ differ at *p* < 0.05.

**Table 6:** Milk antioxidant profile throughout the collection period.

Variable	Sampling Month							SEM	Significance
	March (n = 20)	April (n = 20)	May (n = 20)	June (n = 20)	July (n = 20)	August (n = 20)	September (n = 14)		
TPC (mg GAE/mL) ^1^	1.10 ^a^	1.20 ^a^	1.15 ^a^	1.10 ^a^	1.18 ^a^	1.24	1.39 ^b^	0.406	***
DPPH (μM TE/mL) ^2^	21.10	23.54 ^b,c^	22.99	20.15 ^a,b^	19.41 ^a^	20.89	24.68 ^c^	8.142	**
FRAP (μM TE/mL) ^2^	34.42 ^a^	38.43 ^b^	45.98	37.38	39.24	37.97	34.63	16.850	*
ABTS (μM TE/mL) ^2^	586.95 ^b,c^	490.77 ^a^	505.19 ^a,b^	530.93	507.93 ^a,b^	503.39 ^a,b^	625.86 ^c^	208.197	***

^1^ = total phenolic content; ^2^ = Trolox equivalents (TEs); SEM = standard error of the mean; * = *p* < 0.05; ** = *p* < 0.01; *** = *p* < 0.001; superscripts ^a–c^ differ at *p* < 0.05.

**Table 7:** Changes in physical characteristics during the milk collection period.

Variable	Sampling Month							SEM	Significance
	March (n = 20)	April (n = 20)	May (n = 20)	June (n = 20)	July (n = 20)	August (n = 20)	September (n = 14)		
pH	6.75 ^b^	6.72	6.68 ^a^	6.68 ^a^	6.70	6.72	6.69 ^a^	0.113	***
Electrical conductivity (mS/cm)	5.22 ^a^	5.18 ^a^	5.28 ^a^	5.58 ^b^	5.81 ^b^	5.75 ^b^	5.78 ^b^	1.222	***
Refractive index	1.3479	1.3478	1.3481	1.3481	1.3476 ^a^	1.3482	1.3487 ^b^	0.001	*
Brix (°Bx)	10.06	9.98	10.18	10.21	9.84 ^a^	10.28	10.54 ^b^	0.936	*
Density (g/mL)	1.035	1.035	1.035	1.034	1.035	1.035	1.038	0.005	NS
FDP (−°C)	0.551	0.548	0.543	0.543	0.547	0.551	0.553	0.017	NS

SEM = standard error of the mean; NS = non-significant; * = *p* < 0.05; *** = *p* < 0.001; superscripts ^a,b,^ differ at *p* < 0.054.

## Data Availability

The data presented in this study are contained within the article.
